# Roles of the cGAS-STING signaling pathway in viral latency

**DOI:** 10.1128/jvi.01756-25

**Published:** 2026-03-31

**Authors:** Qirou Wu, Qiongyu Xu, Jie Liu, Tiejun Zhao

**Affiliations:** 1 School of Medicine, Hangzhou City University 728194, Hangzhou, China; 2 College of Pharmaceutical Sciences, Zhejiang University 366096, Hangzhou, China; 3 College of Life Sciences, Zhejiang Normal University 66344, Jinhua, China; New York University Department of Microbiology, New York, New York, USA

**Keywords:** viral latency, cGAS-STING pathway, immune surveillance, viral reactivation, STING agonists

## Abstract

Viral latency is a sophisticated survival strategy that allows the viral genome to persist indefinitely while remaining invisible to immune surveillance. This stealth impedes eradication, and episodic reactivation compounds the hurdle, necessitating therapeutic innovation. Recently, increasing evidence has indicated that the cGAS-STING pathway, which senses nonself or damaged DNA, plays vital roles in the establishment, maintenance, and reversal of latency in a subset of viruses. Given its central role in triggering innate immune responses, the cGAS-STING pathway has emerged as a high-priority target for therapeutic intervention. Here, we outline the multilayered arms race between latent viruses and the cGAS-STING pathway. Latent viruses generally conceal their genomes from cGAS, disrupt cGAS-STING signaling to limit innate immune responses, and subsequently deploy latency-associated factors to maintain pathway silencing and evade immune surveillance. We also elucidated the paradoxical engagement of cGAS-STING during reactivation. Motivated by encouraging preclinical data on STING agonists and the central role of the STING signaling pathway in cell fate decisions, we synthesized the current knowledge and proposed targeting this pathway for therapeutic intervention to reduce viral latency.

## INTRODUCTION

Viral latency is a reversible, transcriptionally silent state that enables long-term persistence of viruses in host cells without the production of infectious progeny. In this state, the viral genome is epigenetically silenced, allowing the virus to evade immune clearance and establish lifelong, covert infection (1). This specialized strategy is employed by only a subset of viruses, such as herpesviruses and some retroviruses (1, 2). For example, human immunodeficiency virus type 1 (HIV-1) integrates its cDNA into host chromosomes to form latent reservoirs in memory T cells (3), whereas Kaposi’s sarcoma-associated herpesvirus (KSHV) forms an episome anchored to host chromosomes (4, 5). Despite mechanistic differences, the latency lifecycle generally comprises establishment, maintenance, and reactivation. Reactivation of latent viruses can lead to episodic viremia, clinical disease, and genomic instability in some cases. Latently infected cells are not susceptible to current antiretroviral drugs or immune clearance. Yet, they harbor proviruses that can reignite systemic infection upon treatment interruption, which is exemplified by HIV-1. In this combination, antiretroviral therapy effectively controls viremia but fails to eliminate the latent reservoir (6). Antiviral therapy has advanced rapidly in recent years. Clinically approved direct-acting antiviral (DAA) combinations have achieved more than 95% functional cure in hepatitis C virus (HCV) infection and are now guiding multitarget therapies for other viruses (7). Moreover, several therapeutic strategies targeting viral latency have shown promise in experimental studies (8, 9). A mechanistic dissection of the molecular circuitry that governs viral latency is, therefore, indispensable for next-generation curative strategies.

**Fig 1:**
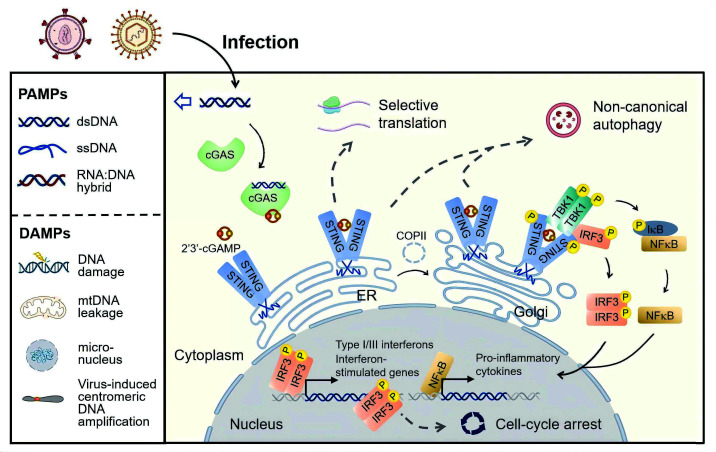
Activation of the cGAS-STING pathway by viruses. During viral infection, PAMPs, including dsDNA, ssDNA, and DNA-RNA hybrids, or DAMPs, such as mitochondrial or nuclear dsDNA released upon stress and virus-induced centromeric DNA amplification, are sensed by cGAS. cGAS catalyzes the synthesis of 2′3′-cGAMP, which binds the ER adaptor protein STING. Following cGAMP binding and conformational activation, STING traffics from the ER to the Golgi via COPII vesicles for downstream signal propagation. Canonically, TBK1-mediated phosphorylation of STING recruits IRF3, leading to IRF3 phosphorylation, dimerization, and nuclear translocation. Together with NF-kB activation, STING drives transcription of IFN and pro-inflammatory cytokines. In addition, non-canonical signalosomes regulate translation, autophagy, and cell-cycle arrest, collectively determining whether infected cells survive or are eliminated.

The therapeutic activation of innate immunity is a broadly effective antiviral strategy and has the potential to reverse latent infection. The cyclic GMP-AMP synthase (cGAS)-stimulator of interferon genes (STING) pathway constitutes the core of this defense. During viral infection, cGAS senses pathogen-associated molecular patterns (PAMPs), such as viral double-stranded DNA (dsDNA), but not viral RNA, as well as damage-associated molecular patterns (DAMPs), such as nuclear or mitochondrial dsDNA released upon cellular stress and virus-induced centromeric DNA amplification (10). Upon binding, cGAS synthesizes the second messenger 2′3′-cyclic GMP-AMP (cGAMP) (11, 12), which activates the endoplasmic reticulum (ER) adaptor protein STING (13, 14). STING then assembles the canonical STING-TANK-binding kinase 1 (TBK1)-interferon regulatory factor 3 (IRF3) signalosome to induce interferon (IFN) activity and trigger nuclear factor kappa B (NF-κB)-dependent production of proinflammatory cytokines (15). STING also initiates noncanonical signaling to restrict viral replication, reprogram transcription (16), and shape cell fate decisions such as apoptosis, senescence, or autophagy (17, 18) (Fig. 1).

While the balance between viral replication and host antiviral responses tips toward the former during the lytic phase, a state of viral-host coexistence prevails during latency, with the cGAS-STING pathway assuming a delicate dynamic equilibrium with the viruses. In terms of persistence, these viruses have evolved multilayered mechanisms to restrict cGAS-STING signaling quantitatively. This evolutionary arms race suggests that finely tuned cGAS-STING engagement has the potential to destabilize viral latency (15). Therefore, a comprehensive understanding of the regulatory and suppressive mechanisms governing the cGAS-STING pathway during latency is essential (19, 20). This review focuses on the interplay between latent viruses and the cGAS-STING pathway during the establishment, maintenance, and reactivation phases of latency. We delineate the viral antagonists that impose sequential blocks at key steps of cGAS-STING signaling and demonstrate how the amplitude of the innate immune response influences the cell fate decisions of host cells. We also outline the mechanistic framework that positions precise modulation of this pathway as a curative strategy for latent infections.

## ROLE OF cGAS-STING AT DIFFERENT PHASES OF VIRAL LATENCY

### Establishment of viral latency

The establishment of latency represents evolutionary adaptation, with the decision to enter latency made during the lytic phase. During this phase, host cells detect viral components and mount innate immune responses to restrict viral replication and eliminate infected cells (21). For instance, during HIV-1 infection, the integration of viral DNA initiates cGAS-dependent signaling and a DNA damage response that together precipitate the senescence and death of some infected CD4^+^ T cells (22, 23). In contrast, many latent viruses encode immunomodulatory proteins that attenuate innate immune signaling, thereby creating a permissive window for genome persistence while preserving host cell viability (24). Although latency-entry strategies differ, increasing evidence indicates that successful establishment of latency requires evolved tactics to inhibit cGAS-STING signaling (20, 25). With a focus on the classical latent viruses, such as HSV-1, HIV-1, Epstein‒Barr virus (EBV), KSHV, human cytomegalovirus (HCMV), and human T-cell leukemia virus type 1 (HTLV-1), we dissect the most recent studies on viral restriction of this pathway, moving stepwise from DNA hiding to signal extinction.

**Fig 2:**
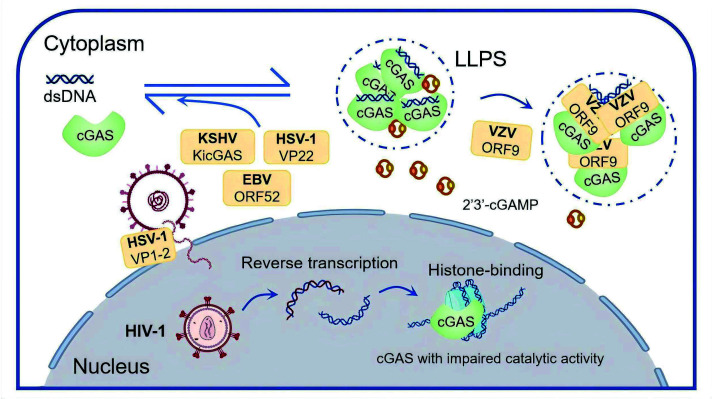
Spatial segregation of cGAS by latent viruses during the lytic cycle. Latent viruses minimize nuclear import transit time and occlude viral DNA from cGAS. HSV-1 anchors incoming capsids at the nuclear pore via VP1-2, limiting cytoplasmic leakage, whereas HIV-1 DNA engages cGAS through a histone-binding surface. In addition, tegument proteins, such as KSHV KicGAS, EBV ORF52, and HSV-1 VP22, disrupt the LLPS of cGAS and dsDNA, and VZV ORF9 interacts with both cGAS and dsDNA in LLPS to inhibit the synthesis of cGAMP.

Spatial segregation of cGAS-STING signaling components is a near-universal innate immune evasion strategy. Although cGAS is best known for sensing cytosolic dsDNA, emerging evidence indicates that nuclear cGAS retains the ability to detect dsDNA under specific conditions. Accordingly, viruses not only minimize the residence time of viral DNA in the cytoplasm but also deploy mechanisms to suppress cGAS activation in both compartments (Fig. 2). HSV-1, for example, tethers incoming capsid to the nuclear pore via the tegument protein VP1-2, reducing cytosolic viral DNA leakage (26). Nuclear cGAS is silenced through high-affinity binding to nucleosomes to prevent aberrant activation by self-genomic DNA (27). During HIV-1 infection, unintegrated HIV-1 DNA is assembled into chromatin-like structures decorated with host histones, which present a platform for cGAS recruitment, leading to its sequestration in a catalytically inactive state (28). In the cytosol, cGAS undergoes liquid‒liquid phase separation (LLPS) upon binding dsDNA to synthesize cGAMP efficiently (29). LLPS is defined as a reversible, membrane-less intracellular compartmentalization process that enables the selective enrichment of biomacromolecules (29–31). Certain viral tegument proteins have been reported to restrict cGAS‒dsDNA phase separation. A prominent example is the KSHV inhibitor of cGAS (KicGAS), which is encoded by ORF52. Compared with cGAS, this protein undergoes DNA-induced phase separation, binds dsDNA with higher affinity, and disrupts preformed cGAS‒dsDNA condensates *in vitro* by extracting dsDNA, thereby suppressing cGAS activation (32, 33). ORF52 homologs in other gammaherpesviruses, such as EBV, also inhibit cGAS activity (34), likely through a similar mechanism. Compared with cGAS, HSV-1 VP22 preferentially forms liquid droplets with DNA, which replace cGAS from the condensation *in vitro* (33). In addition, the Varicella-zoster virus (VZV) ORF9 interacts with cGAS and cophase-separates with dsDNA, thereby sterically limiting the cGAS‒dsDNA interaction and suppressing cGAMP synthesis (35).

Apart from masking viral DNA from cGAS, latency-establishing viruses also enforce spatial segregation of STING. HSV-1 UL38 docks within the cGAMP-binding groove of STING to function as a high-affinity competitive antagonist (36), whereas HCMV UL82 and UL94 tether STING in the endoplasmic reticulum (ER), preventing its COPII-dependent export and the subsequent formation of the STING-TBK1 complex (37, 38). HSV-1 ICP34.5 also disrupts STING translocation from the ER to the Golgi apparatus (39). Human papillomavirus (HPV) 18 E7 binds to STING and selectively suppresses NF-κB activation (40). KSHV ORF48 colocalizes and interacts with STING, thereby suppressing the activation of STING-dependent innate immune signaling (41, 42). KSHV-encoded vIRF1 directly binds STING and competes for TBK1 activation sites, destabilizing the STING-TBK1 complex (42). HCMV UL138 inhibits the pathway downstream of STING but upstream of IRF3 phosphorylation and NF-κB function during lytic infection (43). In addition to antagonizing the canonical STING-TBK1-IRF3 signalosome, HIV-1 Vpu disrupts STING-mediated activation of NF-kB signaling by directly interacting with STING (44). HIV-2/SIV Vpx targets a novel functional domain of STING to inhibit cGAS-STING-mediated NF-κB signaling selectively (45). HSV-1 UL24 selectively inhibits NF-κB promoter activation without affecting IRF3 activation (46). These examples underscore the selective modulation of innate signaling by a viral accessory protein.

Targeted modification or degradation of cGAS-STING pathway components represents another commonly employed viral strategy. HSV-1 provides a clear example. UL41 harnesses its intrinsic RNase activity to degrade cGAS mRNA (47). UL37 possesses deamidase activity that selectively modifies human and murine cGAS to decrease its ability to synthesize cGAMP (48). UL36USP deubiquitinates IκBα, preventing its degradation and thereby inhibiting NF-κB activation (49, 50). Similarly, HSV-1 VP22 and HCMV UL31 also inhibit the enzymatic activity of cGAS (51, 52). With respect to STING, EBV BPLF1 associates with STING and acts as an effective deubiquitinating enzyme that removes K63-, K48-, and K27-linked ubiquitin chains from STING and strips ubiquitin from TBK1 kinase, leading to the protein degradation (53). Viral polymerase (Pol) of hepatitis B virus (HBV) reduces K63-linked polyubiquitination of STING (54). HCMV pUL48 deubiquitinates STING to silence IFN signaling and promote tumorigenesis (55). In addition, the STING-downstream transcription factor IRF3 is subject to modulation by viral proteins, such as HSV-2 ICP27, HHV-6 IE1, and HSV-1 VP24, altering its posttranslational modification and subcellular trafficking (56–58).

In addition to direct interactions and modification, viral proteins also subvert the cGAS-STING pathway by targeting essential host cofactors. For instance, HSV-1-induced AKT and DNA-dependent protein kinase (DNA-PK) phosphorylate cGAS, and both events collectively impair cGAS catalytic activity (59, 60). HTLV-1 Tax recruits host USP20 to remove K63-linked ubiquitin chains from STING, thereby blocking the formation of the STING-TBK1 complex (61). Virus-activated host phosphatases, such as PPM1A and PTPN1/2, dephosphorylate STING, blocking TBK1 recruitment to STING and inhibiting signal transmission (62, 63). Additionally, *β*-catenin enhances IFN-I transcription in the cGAS-STING pathway, and HSV-1 counteracts this host defense by hyperphosphorylating *β*-catenin via its kinase US3, thereby inhibiting IFN-I production and evading innate immunity (64). Collectively, these examples illustrate that many latent viruses, ranging from herpesviruses to retroviruses, have evolved distinct yet convergent strategies to suppress the cGAS-STING pathway at multiple levels during the establishment of latency.

### Maintenance of viral latency

Despite global transcriptional silencing, latency is not an absolute state of dormancy. A select repertoire of latency-associated viral proteins continues to be expressed and exhibits functional pleiotropy in orchestrating the molecular mechanisms of viral persistence (1). Importantly, a growing body of evidence indicates that a diverse array of latency-associated proteins encoded by different viruses exert regulatory effects on the cGAS-STING pathway. These findings further highlight the pivotal role of the pathway in viral latency, as its effective suppression is indispensable for viruses to evade host detection and maintain long-term persistence.

One of the most intensively studied latency-associated proteins in tumor virology is the latency-associated nuclear antigen (LANA) of KSHV. LANA is among the most abundantly expressed viral proteins during latency (65) and is consistently present in all KSHV-associated tumor cells (66). Full-length LANA resides in the nucleus, where it tethers the circular KSHV episome to host chromatin, restricting episomal replication and ensuring equal segregation during mitosis (67). Loss of LANA triggers rapid clearance of viral DNA through a cGAS-dependent autophagic pathway, demonstrating that episome concealment is an essential immune-evasion function of the protein (68). N-terminally truncated cytoplasmic isoforms of LANA bind cGAS, preventing dsDNA recognition and LLPS, and they also colocalize with STING, remove K63-linked ubiquitin chains from activated STING, and impair the assembly of the STING-TBK1-IRF3 complex (69). LANA has also been shown to compete with IRF3 for binding to the IFNβ promoter, thereby directly suppressing IFN-I transcription (70). Additionally, LANA promotes cell cycle progression by antagonizing Rb-mediated cell cycle arrest, thereby facilitating S phase entry (71), which may bypass STING-IRF3-pRb axis-mediated cellular senescence. Similarly, g-herpesviruses employ functional LANA counterparts, such as EBV EBNA1, MHV-68 ORF73, and HVS ORF73, to antagonize innate and adaptive immunity and increase latency (72). The HTLV-1 latency-associated protein HBZ has also been shown to suppress IRF3 activation (73). With respect to EBV latency, latent membrane protein 1 (LMP1) has recently been shown to trigger the deregulation of the cGAS-STING pathway (74).

Beyond the strictly latency-restricted proteins, certain viral factors persist in both the lytic and latent phases. Once viruses commit to latency, viral proteins may undergo rewiring of their subcellular localization, posttranslational signature, and absolute abundance, enabling them to limit host anti-viral signals. HCMV provides two well-documented examples. During lytic replication, US9 accumulates in the ER-Golgi intermediate compartment (ERGIC) where it blocks STING trafficking, but is redistributed to ERGIC-53-positive vesicles during latency. This relocation specifically prevents STING dimerization and aborts downstream IFN signaling without altering total STING abundance (75). Likewise, UL138, a classical latency-associated determinant of HCMV, interacts with both STING and TBK1, inhibiting IRF3 phosphorylation during latency within incompletely differentiated myeloid cells (43). KSHV vIRF1 is readily detected in latent B-cell lines, and although its abundance is lower than that during lytic replication, this residual pool is sufficient to disrupt the TBK1‒STING interaction and block IRF3 phosphorylation (42).

Collectively, these findings illustrate that the maintenance of viral latency does not simply involve the silencing of lytic-phase antagonists. Instead, viruses establish latency-suited shields against the cGAS-STING pathway. The function of these latency-associated proteins highlights the cGAS-STING pathway as an important barrier whose neutralization is essential for the long-term persistence of specific viruses (1, 70).

### Reactivation of the latent virus

After a period of latency, the virus can re-enter the lytic cycle in a process known as reactivation. Reactivation is typically triggered by various factors, including self-periodic viral modulation, stress responses, immunosuppression, or changes in the physiological state of infected cells (24). Like lytic replication, viral reactivation is associated with increased levels of dsDNA derived from viral genomes or infection-induced DNA damage, which can activate the cGAS-STING pathway (76, 77). Accordingly, silencing cGAS or STING markedly augments the reactivation of latent viruses, such as KSHV (42, 69).

Although the catalytic pocket of cGAS is normally masked by densely packed histones and silenced within the nucleus, recent works have shown that the host senses viral reactivation far more rapidly than anticipated. In addition to detecting pathogen DNA directly, cGAS can detect infection through a pathway termed virus-induced centromeric DNA amplification and recognition (VICAR) (10). Latent HSV-1 genomes reside adjacent to centromeres; upon reactivation, the immediate-early protein ICP0 targets and degrades centromeric proteins, disrupting their integrity (78). This action inadvertently engages the trans-lesion synthesis (TLS) machinery, which amplifies centromeric repeats, generating naked, histone-poor self-DNA fragments that activate nuclear cGAS. In HSV-1-infected mouse macrophages, approximately one-third of total cGAMP is produced via the ICP0-TLS axis (10). However, ICP0 also upregulates corneal suppressor of cytokine signaling 1 (SOCS1) and SOCS3 expression, which inhibits STING-induced IFN signaling (79). Like ICP0, the immediate-early 1 (IE1) protein of cytomegalovirus (CMV) also induces centromeric DNA amplification and cGAS activation (10). Notably, cGAS-STING signaling involves intercellular transmission (80), and the viral proteins can also be transferred between cells via extracellular vesicles (EVs). Following reactivation in a subset of nasopharyngeal carcinoma (NPC) cells, latent EBV expresses its encoded BRRF2, which is secreted via EVs and specifically targets macrophages. Mechanistically, BRRF2 disrupts cGAS binding to dsDNA and inhibits cGAS‒dsDNA phase separation, thereby attenuating cGAS enzymatic activity (81). Viruses have also evolved multiple strategies to evade or suppress the cGAS-STING pathway during reactivation, similar to those observed during primary infection. Upon reactivation in B cells, EBV selectively degrades the genome-guarding cohesin SMC5/6, thereby shielding viral DNA from cGAS surveillance (82). Additionally, the UL37 protein of HSV-1 has been shown to suppress the cGAS-STING signaling pathway, thereby facilitating viral reactivation (48). These findings advance the understanding of immune evasion and unlock novel targeted strategies to improve immunotherapy efficacy.

Interestingly, immune evasion is driven not only by viral proteins but also by microRNAs. Three KSHV miRNAs, miR-K12-6-3p, miR-K12-7-3p, and miR-K12-11-3p, directly bind to *STING1* mRNA, repressing its translation and blocking innate immune signaling. These miRNAs are essential for viral reactivation, as their genetic deletion upregulated STING protein levels in latent KSHV cell lines and delayed lytic reactivation (83).

**Fig 3:**
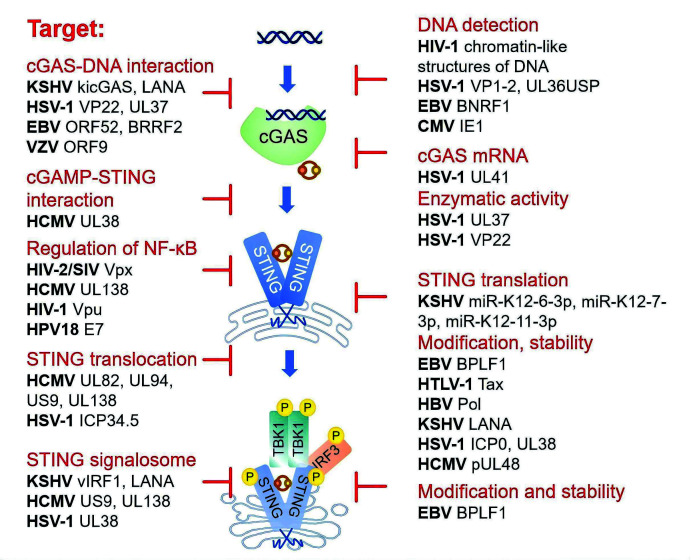
Recent insights into the molecular mechanisms underlying suppression of the cGAS-STING-TBK1 axis by latent viruses. Schematic summary of recent strategies reported in latent viruses like HSV-1, EBV, KSHV, HCMV, HBV, and HTLV-1 to inhibit the cGAS-STING-TBK1 axis, including spatial segregation of pathway components, targeted modification and degradation of key factors, and hijacking of host regulatory proteins. These multilayered mechanisms enable viral immune evasion and maintenance of latent viruses by dampening innate immune responses.

In summary, the cGAS-STING pathway serves as a central innate immune checkpoint that mediates the dynamic interplay between latent viruses and host cells across the establishment, maintenance, and reactivation phases (Fig. 3). Understanding the interplay between the host cGAS-STING pathway and viral reactivation is crucial for elucidating the mechanisms of viral latency and for developing targeted strategies against latent infections.

## THERAPEUTIC PROSPECTS OF TARGETING cGAS-STING IN LATENT VIRAL INFECTIONS

The cGAS-STING pathway serves as a key mediator of innate immunity and plays critical roles in the pathogenesis of diverse diseases, including viral infections, tissue fibrosis, and cancer (18). In recent years, STING agonists have emerged as highly promising immunotherapeutic agents (Table 1). For example, the chemical agonist GNE-6468 potently triggers both STING-dependent innate immunity and noncanonical autophagy, resulting in robust anti-HSV-1 activity (84). Innovative delivery strategies that self-assemble selectively within tumor lesions have been developed to spatially restrict STING activation (85). Notably, many chronic viral infections drive organ fibrosis and initiate oncogenesis (86). In this context, STING agonists not only mitigate virus-induced tissue injury and fibrotic remodeling (87) but also have strong potential for application in cancer immunotherapy (88). STING agonists, such as diABZI, demonstrate the most potent, broad-spectrum antiviral function (89), and many recent works have leveraged SARS-CoV-2 infection models. STING agonist-based ER-targeting molecules (SARBs) efficiently deliver antigens to the ER. Conjugation of SABER enhances CD8^+^ T-cell immune responses against conserved SARS-CoV-2 epitopes (90). Nanoformulated STING agonists (nanoSTING) have been demonstrated to be effective at preventing SARS-CoV-2 reinfection (91), whereas the systemic activation of STING with small-molecule agonists, such as diABZI or MSA-2, provides strong protection against primary SARS-CoV-2 challenge (92, 93).

**TABLE 1:** STING agonists in development

Compound	Type	Stage	Disease model	Reference or clinical trial identifier
GNE-6468	Transmembrane domain-targeting agonist	Pre-clinical	HSV-1 infection, MC38 tumor model	(84)
MSA-2	Oral benzothiophene small molecule	Pre-clinical	Murine tumor models	(94)
SR-717	Non-nucleotide agonist	Pre-clinical	Murine tumor models; radiation experiment	(95, 96)
IACS-8803 and IACS-8779	CDN analog	Pre-clinical	B16 murine model	(97)
M335	CDN analog	Pre-clinical	HSV-1 infection, murine tumor models	(98)
exoSTING	Exosome-delivery system	Pre-clinical	Murine tumor models	(99)
diABZI	STING-activating benzothiazole	Pre-clinical	Murine tumor models; viral infection models (SARS-CoV-2, influenza, HBV, etc.)	(89, 100–104)
ONM-501	Micellar nanoparticle	Phase I	Advanced solid tumors	NCT06022029
HG-381	Small-molecule non-CDN	Phase I	Advanced solid tumors	NCT04998422
XMT-2056	Antibody-drug conjugate (HER2)	Phase I	HER2-expressing solid tumors	NCT05514717
GSK3745417	Small-molecule non-CDN	Phase I	Advanced solid tumors	NCT05424380
SNX281	Small-molecule non-CDN	Phase I	Advanced solid tumors/lymphoma	NCT04609579
TAK-676	CDN analog	Phase I/II	Solid tumors	NCT04879849
CDK-002	Pro-drug of CDN analog	Phase I/II	Solid tumors	NCT04592484
DN-015089	CDN analog	Phase I	Advanced solid tumors	CTR20212462
KL340399	Small-molecule non-CDN	Phase I	Advanced solid tumors	NCT05549804
ONO-7914	Nanoparticle-encapsulated agonist	Phase I	Advanced solid tumors	jRCT2031210530
MK-1454	CDN analog	Phase II	Solid tumors	NCT04220866
MK-2118	CDN analog	Phase I	Solid tumors	NCT03249792

Moreover, the canonical STING agonist cGAMP has been incorporated into the "shock and kill" strategy for targeting latent viruses (19, 105). This strategy involves two sequential steps: the reactivation of latent viruses by latency-reversing agents (LRAs) and the subsequent elimination of infected cells through viral cytopathic effects or immune-mediated clearance (106). Originally devised for HIV eradication, the "shock and kill" strategy has now been extended to other latency-associated viruses, such as KSHV and EBV (107, 108). Interestingly, the cGAS-STING pathway plays a dual role in this process. The suppression of cGAS-STING can promote the reactivation of latent viruses, whereas STING-mediated cell death serves as an efficient mechanism for the clearance (109). The canonical STING agonist cGAMP has been incorporated into the "shock and kill" strategy. The combination of cGAMP with the histone deacetylase (HDAC) inhibitor resminostat can induce reactivation and HIV-infected T-cell death in cell lines, primary T lymphocytes, and patient samples (19). Specifically, in CD4^+^ T cells from individuals on suppressive antiretroviral therapy (ART), this regimen can still reduce total viral DNA, indicating substantial reservoir contraction (19). Concurrently, HIV-1-specific CD8^+^ T cells are considered the principal effectors of reservoir clearance. Strikingly, cGAMP efficiently primes naive T cells to generate CD8^+^ T lymphocytes that are specific for protective HIV-1 epitopes (110). These cells exhibit high expression of cytolytic effector molecules and robustly suppress viral replication, offering a novel strategic avenue toward a functional cure for HIV-1.

Beyond initiating canonical inflammation, STING context-dependently triggers noncanonical autophagy and programmed cell death (109), which provide multiple effector modules for eradicating latent pathogens. For instance, STING activates transcription factor EB (TFEB) via γ-aminobutyric acid receptor-associated protein (GABARAP) lipidation, markedly increasing lysosomal biogenesis and accelerating the clearance of cytosolic DNA and multiple pathogens (111, 112). STING-induced noncanonical autophagy effectively degrades HSV-1 via the lysosomal pathway in cell-based models (105), indicating that STING is an efficient antiviral route for host cells.

Notably, on the basis of the detailed molecular mechanisms by which virus-encoded factors inhibit the cGAS-STING pathway, researchers have developed competitive occupancy strategies for targeted drug development (113, 114). With respect to HSV-1 infection, L4P, a competitive peptide designed on the basis of the sequence of the key binding domain of STING, exhibits extremely high binding affinity to the viral antagonist protein UL38 to restore the binding capacity of STING to cGAMP, significantly increasing the survival rate of mice with HSV-1 encephalitis (36, 113). Intriguingly, the representative cyclopeptide XQ2B docks at the cGAS-dsDNA interface and disrupts LLPS, blunting HSV-1-elicited antiviral responses and dampening cGAS-driven inflammatory cascades (114). Viral proteins, such as KSHV LANA and VZV ORF9, also interact with cGAS (35, 69), underscoring their potential as structural scaffolds or mechanistic blueprints for next-generation cGAS-STING antagonists.

Collectively, these findings indicate that STING agonists can assist in clearing actively replicating viruses and, by orchestrating innate immune responses, noncanonical autophagy, and programmed cell death, can also force latent viruses out of hiding and elimination. Nevertheless, only a handful of these agonists have been evaluated in viral latency models, and even fewer have undergone rigorous validation in *in vivo* or early-phase human studies. Consequently, the therapeutic landscape for STING-targeted interventions against latent infections remains largely untapped, representing a promising avenue for future research.

## CONCLUSION

The cGAS-STING pathway plays a pivotal and complex role across the establishment, maintenance, and reactivation phases of viral latency. On the one hand, activation of this pathway induces innate immune responses and determines the cell fate of infected cells, thereby restricting the persistence of latent viruses. On the other hand, viruses employ multiple mechanisms to suppress this pathway for immune evasion and maintenance of latency (115). STING-targeted agents, whether they are cyclic dinucleotide (CDN) analogs, pro-drugs, immune-stimulating antibody conjugates (ADCs), or competitive peptides, hold the potential to restrict viral persistence across latency phases, yet their clinical translation remains a still-emerging field.

Looking forward, the bench-to-clinic translation faces a steep hierarchy of unresolved challenges. In terms of precision targeting, latently infected cells may be vanishingly rare, anatomically dispersed, and sequestered within immune-privileged sanctuaries, such as CD4^+^ central memory T cells, neuronal ganglia, and B-cell follicles (116). Their unambiguous identification will, therefore, require unique surface or metabolic signatures that can be exploited for selective delivery. Short-pulse "shock" strategies may transiently awaken the reservoir (106) and phenotypically distinguish it from uninfected cells. Moreover, prolonged STING signaling, akin to that observed in tumor models, can promote an immunosuppressive microenvironment (117) that ultimately blunts clearance and may drive systemic IFN-mediated toxicity (118). Hence, delivery modalities, such as EVs, ligand-display nanoparticles, or other targeted nanocarriers, are indispensable for the confinement of STING analogs to the intended cells (80). Temporal control within the viral latency cycle is equally critical. High-resolution, single-cell and spatial transcriptomics may, therefore, be leveraged to chart the latency clock in each tissue, enabling on-off dosing schedules that synchronize proviral reactivation, antigen presentation, and effector entry while minimizing bystander inflammation (119). Furthermore, the viral mechanisms of cGAS-STING regulation offer a blueprint for designing STING inhibitors to curb excessive inflammation and cytokine storms.
